# The need for European OneHealth/EcoHealth networks

**DOI:** 10.1186/s13690-017-0232-6

**Published:** 2017-10-26

**Authors:** Hans Keune, Lucette Flandroy, Séverine Thys, Nick De Regge, Marcella Mori, Nicolas Antoine-Moussiaux, Maarten P.M. Vanhove, Javiera Rebolledo, Steven Van Gucht, Isra Deblauwe, Wim Hiemstra, Barbara Häsler, Aurélie Binot, Sara Savic, Simon R. Ruegg, Sjerp De Vries, Julie Garnier, Thierry van den Berg

**Affiliations:** 1 Belgian Community of Practice Biodiversity & Health (COPBH), Brussels, Belgium; 2 Belgian Biodiversity Platform, Brussels, Belgium; 3 Research Institute Nature & Forest (INBO), Kliniekstraat 25, 1070 Brussels, Belgium; 4 University of Antwerp, Faculty of Medicine and Health Sciences - Drie Eiken, gebouw R R.3.07, Universiteitsplein 1, 2610 Wilrijk, Belgium; 5 Federal Public Service Health, Food Chain Safety and Environment – DG Environment, Victor Horta Square, 40, box, 10, 1060 Brussels, Belgium; 6 Institute of Tropical Medicine of Antwerp (ITM), Nationalestraat 155, 2000 Antwerp, Belgium; 7 CODA-CERVA, Groeselenberg 99, 1180 Brussels, Belgium; 8 University of Liège, Faculty of Veterinary Medicine (ULiege), 6 avenue de Cureghem, 4000 Liège, Belgium; 9 Capacities for Biodiversity and Sustainable Development (CEBioS), Operational Directorate Natural Environment, Royal Belgian Institute of Natural Sciences, Vautierstraat 29, 1000 Brussels, Belgium; 10 Department of Botany and Zoology, Faculty of Science, Masaryk University, Kotlářská 2, 611 37 Brno, Czech Republic; 11 Laboratory of Biodiversity and Evolutionary Genomics, Department of Biology, University of Leuven, Charles Debériotstraat 32, 3000 Leuven, Belgium; 12 Hasselt University, Centre for Environmental Sciences, Research Group Zoology: Biodiversity & Toxicology, Agoralaan Gebouw D, 3590 Diepenbeek, Belgium; 13 Scientific Institute of Public Health (WIV-ISP), Juliette Wytsmanstraat 14, 1050 Brussels, Belgium; 14 Dutch Farm Experience/Natural Livestock Farming, Winklerlaan 8, 3571 KJ Utrecht, Utrecht, Netherlands; 15 Royal Veterinary College, Hawkshead Lane, Hatfield, AL9 7TA UK; 16 French Agricultural Research and International Cooperation Organization (CIRAD) - UMR 117 ASTRE – Campus International de Baillarguet, 34398 Montpellier, France; 17 Scientific Veterinary Institute “Novi Sad”, Rumenački put 20, Novi Sad, 21 000 Serbia; 18 Vetsuisse Faculty University of Zurich, Winterthurerstrasse 270, 8057 Zürich, Switzerland; 19 Wageningen University and Research, Box 47, 6700AA Wageningen, Wageningen, PO Netherlands; 20 Odyssey Conservation Trust, Bakewell, DE45 1LA Derbyshire UK

**Keywords:** One Health, EcoHealth, Community of Practice, Europe, Interdisciplinarity, Transdisciplinarity, Cross-sectorial

## Abstract

Elaborating from the European One Health/Ecohealth (OH/EH) workshop that took place in fall 2016 and aimed to bring together different communities and explore collaborative potential, the creation of European networks focusing on the development of important OH/EH perspectives was a direct output from discussions at the end of some sessions, in particular:

- A network on transdisciplinary One Health education.

- A network integrating inputs from social sciences in One Health/EcoHealth actions and networks.

- A network aiming at translating research findings on the Environment-Microbiome-Health axis into policy making, with a view to make healthy ecosystems a cost-effective disease prevention healthcare strategy.

It was also suggested that a European Community of Practice could be initiated in order to support these several concrete networking initiatives, and to help to promote the building of other emerging initiatives.

## Background

The importance of human health linkages with nature and the environment in general has been gaining attention in science, policy and society at large. The recent “State of Knowledge” review *Connecting Global Priorities: Biodiversity and Human Health,* co-led by the Convention on Biological Diversity (CBD) and the World Health Organization (WHO) [1], provides a comprehensive overview of the diversity and complexity of interactions between biodiversity and human health and examine related opportunities and challenges at the science-policy interface. In order to address these interrelated aspects of the natural world and human health in a more integrated and holistic manner, several frameworks have emerged over time. The WHO-CBD *State of Knowledge Review* draws on integrative approaches such as One Health (OH), EcoHealth (EH) and Planetary Health. These approaches explicitly consider impacts on human, animal and ecosystem health and their connectivity and are closely aligned with the ecosystem approach, which is the primary framework for action under the Convention on Biodiversity. Several key messages from these initiatives - tailored to the scientific and policy communities and society at large - promote a holistic approach such as OH as an integrative framework for addressing the science-policy challenges at the human-animal-ecosystem health interface. As another initiative at the international governance level, the FAO–OIE–WHO Tripartite Concept Note, ‘*The FAO–OIE–WHO Collaboration – Sharing responsibilities and coordinating global activities to address health risks at the animal–human–ecosystems interfaces*,’ [2] paved the way towards an increasingly integrated OH approach that incorporates a collaborative, cross-sectorial, multidisciplinary perspective on preventing, reducing and mitigating health risks at the human/animal/environment interface.

CBD decision XII/21 on biodiversity and human health recognizes the relevance of the linkages between biodiversity and human health for the 2030 Agenda for Sustainable Development and the Sustainable Development Goals. In this context, CBD invites all concerned Parties and relevant stakeholders to consider the findings of the *State of Knowledge Review* to identify opportunities for supporting national strategies, action plans and programmes on biodiversity that are mutually supportive to those on health. The decision also recognized the value of the OH approach to address the crosscutting issue of biodiversity and human health. A recommendation made at the nineteenth meeting of the Subsidiary Body on Scientific, Technical and Technological Advice of the CBD (SBSTTA) further emphasized the value of this approach. In line with this decision, parties, including European governments, are invited to contribute to and report on their progress towards integrating human, animal and environmental health strategies, as a contribution to the achievement of the Aichi Biodiversity Strategy 2011–2020, Aichi Biodiversity Target 14 and related targets [3]. More recently, the feasibility of using an OH approach towards achieving the Sustainable Development Goals of the 2030 Agenda was reviewed [4].

The European OneHealth/EcoHealth (OH/EH) workshop [5] aimed at facilitating reflection, exchange, mapping future avenues and supporting collaboration of working on the linkages of biodiversity and human health, or linkages within an OH framework. Given the similarities in their objectives to create synergies between health benefits for humans, animals and the environment, the OH and EH concepts appear to be supported by converging communities, working towards a shift from narrow and restricted framings towards systems approaches. Whilst having different origins – EH stemming more from a sustainable health action research perspective, OH more from a human and animal health expert collaboration perspective –, the two approaches converge in emphasizing *“a holistic understanding of health beyond the purely biomedical”* and championing *“systems thinking as a way of achieving a greater understanding of health problems, and both espouse inter- and trans-disciplinary research and collaborative participation”* [6]. The general objective of the workshop was to foster collaboration between OH/EH and related concepts and communities that endeavour to combine ecosystem, animal and human health, and to build bridges between science, policy and practice active in the domain of nature and health. This commentary provides a synthesis of the challenges that were discussed during the workshop as well as the next practical steps that were identified, one of the general highlighted outcomes of the workshop being the emerging need for European OH/EH networks, such as Communities of Practice. A Community of Practice is a network made up of individuals and organizations that share an interest and practice, who come together to address a specific challenge, and further each other’s goals and objectives in a specific topic area [7, 8].

### Workshop format

Over a hundred experts from different professional backgrounds (science, policy & practice) and different fields of expertise contributed to the workshop. They included natural scientists, animal and human health scientists as well as social scientists, policy representatives from national governments and the EU, and experts working in Europe, but also in other regions in the world. For an overview of the various fields of expertise represented in the workshop, please see the participants list [5]. The workshop program featured a combination of specific topics and generic integrative sessions. PowerPoint presentations of the sessions, containing dedicated references can be reached through the Sessions webpage of the workshop (http://www.biodiversity.be/health/82).

In the topical sessions, participants exchanged experiences and views from their fields and projects, whilst exposing the arguments for and possible ways to apply the One Health perspective in their areas of expertise. Such a broad range of issues was selected in order to reflect the diversity of thematic areas presented in the CBD – WHO *State of Knowledge Review* as well as the cross-sectorial and interdisciplinary challenges faced by the OH community. It was noted by participants that such a wide array of cross-sectoral issues was not common in expert meetings: e.g. biodiversity related issues are usually less featured in discussions of the OH community, and expert communities that tackle health benefits from nature contact or experience do not often engage with communities focussing on nature related health risks such as infectious diseases. One follow-up of the workshop, in an OH/EH perspective, should be to deepen links existing between the issues tackled in and the outcomes from these different sessions (see p. 16 and 19 of the workshop report, in ref. [5]). More generic sessions followed on 1. Evaluation and challenges/limitations of OH, 2. Social science, transdisciplinarity and traditional knowledge systems, 3. OH/EH in the Global South: interdisciplinarity building in research and educational challenges.

### Vectors and vector-borne diseases

This session focussed on vectors and vector-borne diseases (VBD), and the complex transmission cycle used by some viruses, bacteria and parasites, involving different vector species and various hosts for their survival, reproduction and spread. The pathogens responsible for VBD are transferred between hosts by arthropod vectors. Current environmental changes linked to human activities (a.o. climate change, landscape changes) together with increased globalization and the use of antimicrobial products and insecticides can rapidly change the distribution, composition, abundance and dynamics of pathogens and vectors. This can result in changes at the pathogen - vector - host interface and could potentially be accompanied by changes in host spectrum and pathogen virulence.

This session highlighted that surveillance and control of vectors and vector-borne diseases is a very broad, complex and multi-disciplinary domain, since all vectors and diseases have their own peculiarities and no unique solution is available to tackle all of them. Therefore researchers should try to define priorities more clearly and work together with policy makers to define clear goals for surveillance activities. Policy makers are therefore advised to act pro-actively by installing surveillance and action/control plans and stimulate implementation of diagnostics for VBD and studies on vector-host-pathogen interactions, and not to wait for an autochthonous spread of vector-borne diseases to react. Given the complexity and multi-disciplinary character of this field, it was furthermore highlighted that this upcoming threat can only be tackled efficiently by an improved collaboration and communication between all different stakeholders like medical doctors, veterinarians, entomologists, researchers, environmental agencies and policy makers.

### Zoonotic diseases

This session covered different topics related to (non-food borne) zoonotic agents that are transmitted under natural circumstances from vertebrate animals to humans. Since historical times, human beings have raised animals for food or kept them in homes as companion pets. The increase in movements of people, and in trade of animals and animal products have accounted with time for the spread and re-emergence of old or new zoonoses. In this particular session, the scientific developments for some important zoonoses were discussed to tackle the OH concept of zoonotic diseases from three perspectives (http://www.biodiversity.be/health/71). A general overview aimed at summarizing zoonotic diseases important for Europe and the rest of the world, by providing examples on specific pathogens and integrating analyses on specific drivers. A surveillance part overviewed the current surveillance initiatives present at the Belgian level (taken as an example) to survey for emergence of zoonotic diseases in humans and domestic production animals. Finally, a control part provided insights on past and ongoing control programmes to fight against zoonotic diseases with an emphasis on success and pitfalls. An initial issue concerned semantics of zoonotic diseases (diseases of animal origin or contracted from animal as cause or human-induced as consequence) and identification of what is necessary to tailor and tackle proper needs that, once identified, will lead to a targeted scale of action. However, prioritization of diseases (for instance on basis of their pandemic potential) is an option explored currently by the stakeholders but it is not shared as the best option within the scientific group participating in the discussion. The idea of working in networks is necessary, including the involvement of the public, and using a common language, in particular because communication of results of research on zoonoses are often jeopardized by institutional/private/country interests. It is important to try to work on a proactive/preventive early warning system of detection rather than a reactive one, when new health issues have already risen. On the other hand, enzootic diseases (like some neglected zoonoses) are present in the animal reservoirs for ages and an early warning system could help learning more about the environmental disturbances at the origin of a modified transmission of these diseases. In any case, more support should be devoted to a better surveillance in order to characterize disease and assess which control measures would be effective and economically efficient, together with initiatives to understand the biology of pathogens, their ecology, the relationship of the pathogen with the host and the host immune responses.

### Agro-eco-human health perspectives on reduction of antimicrobial resistance

The session focused on the global threat of antimicrobial resistance (AMR), which represents an important challenge for human, animal and environmental health experts and practitioners to overcome disciplinary silos and speed up understanding and action towards an OH/EH approach and practice. The session highlighted some priority actions to better manage AMR in an OH approach like: 1. set up research programs in order to conduct in-depth stakeholders analysis (as the interactions diagram that was co-designed during the workshop showed that stakeholders were very diverse and did not fit in “generic boxes”, thus necessitating in-depth analysis of the specific stakes at play, their practices and social networking); 2. support participatory learning, research and innovation in the livestock sector, including private sector partners, that combine farm level improvement (animal management, strategic use of local breeds, use of medicinal plants) with quality control systems and extra payment for residue-free products, that support and validate practice-proven alternatives to reduce antimicrobial resistance developed by (traditional knowledge holders in) farming communities. 3. identify and document processes at play: resistance, mutation, immunity / resilience / trade / communication, education, knowledge sharing / regulation; 4. strengthen the environmental dimension at agro-ecosystem level in AMR management to better understand socio-ecosystem’s dynamics, farm level improvements, ecological functions and services involved in the regulation of resistance and to better understand actors’ practices and socioeconomics rationales; 5. improve data collection, management and sharing to increase interoperability and 6. strengthen networking (science policy interface, university alumni, OH students’ networks, networking between researchers, decision makers and civil society to better address societal demand).

### Environmental and internal microbiome

While the microbial diversity has been recognized as an important ecosystem service making the link between biodiversity and human health [9], it is seldom or never tackled as such in OH or EH fora till now. Yet, beside their effects in infectious diseases and in natural cycles of elements, myriads of microbes inhabit all forms of macro-organisms in which they play crucial physiological roles. The composition of these symbiotic and commensal microbiota are in constant dialogue and interchange with the microbes of the surrounding environment, making of this microbial world an essential interconnecting living network. In an EH perspective, one may thus easily understand that factors affecting the microbiota in one compartment could impact the health of other compartments of the global ecosystem.

The session highlighted the growing scientific evidence explaining why human gut and respiratory tract microbiome disturbance could lead to increasingly occurring non-communicable diseases having inflammation as a common pathological characteristic. A high biodiversity was underlined among common features between microbiota of plants, soils, and animals, including humans, as having positive impacts on their respective health, consistently with the *biodiversity hypothesis* or *old friends hypothesis*. Noteworthy, data in animals and humans suggest transgenerational impacts on the health of their progeny through epigenetic effects of their microbiota. However, while knowledge is in progress on these aspects, a lot of research is still needed in order to unravel the respective roles of genetics and environment, age, different foods, diets, additives, drugs, agricultural systems, general surrounding, various chemical substances and combined effects of these, on the composition and related functions of our microbiome and consequent impacts on our health, as well as in order to better precise the correlations between microbiota of humans, animals, plants/soil and the general environment. Moreover, external factors like chemical substances could affect simultaneously interacting microbiota of humans, animals, plants, surrounding environment, leading potentially to amplified impacts. The session thus concluded on the necessity of more interdisciplinary research to elucidate those various influences and interactions and their consequences on our health and that of the environment. Integration of different concerned policy sectors (health, environment, agriculture, food security, land use planning, housing) should favour financing such kind of research. Their results could lead to necessary reviewing legal risk assessment requirements for some compounds.

To favour the development of such interdisciplinary research and the translation of research findings into integrated policies, the session proposed the creation of a European science-policy platform where the microbiome would constitute a leading element and health indicator interconnecting various compartments of the global ecosystem and thus various European policies; ensuring by that way healthy ecosystems could constitute a cost-effective preventive healthcare strategy, in an EH approach integrating various societal decisions and Sustainable Development Goals of the UN 2030 Agenda. In any case, exposure to high microbial biodiversity has now been shown to be correlated to the overall positive health impacts of living close to natural environments (link with next session); this current knowledge is thus supporting reasons for promoting access to and contact with nature, with high biodiversity, in particular in urban areas, where no obvious risks of infectious diseases are known.

### Nature health benefits

The session focussed on the many ways nature benefits human health. Examples highlighted the importance of nature (and often its biodiversity) to traditional and modern medicinal practice, and the utility of various species for medical research. Genetic and species diversity is functional to food production, and plays an important role in nutrition security by potentially reducing risks of non-communicable diseases often linked with poor nutritional quality (e.g. obesity, diabetes, micronutrient deficiencies) [10]. Nature also plays a role in safeguarding air quality and access to freshwater, can help mitigating disaster risk, while supporting emergency responses and climate change adaptation. Furthermore, diverse natural environments may enhance experiences that reduce stress, support the development of cognitive resources, stimulate social contacts, attract people to physical activity, and support personal development throughout an individual’s lifespan. Moreover, recent studies [11–13] show that declining contact with some forms of (microbiotic) life may contribute to the rapidly increasing prevalence of allergies and other chronic inflammatory diseases among urban populations worldwide (cf. previous session). Access to nature therefore can make an important contribution to both public health related ecosystem services and to the reduction of health risks. The session discussed a diversity of experiences, expectations, opportunities and challenges regarding nature health benefits in science, policy and practice. It also highlighted the difficulties of providing strong evidence of nature health benefits. At the same time it was felt that practice should not wait for perfect science, especially since nature-based solutions to health problems in general are likely to have trade-off side-effects. For example, health benefits from urban parks due to increased physical and social activity can go hand in hand with increase of pollen allergies or risk of tick-borne diseases [14]. All participants recognized the limitations of economic valuations for the complex natural, social and economic systems involved, despite their popularity among policy makers who need to make trade-offs.

### Evaluations and challenges/limitations of one health

Worldwide recognition of OH for more effective protection of animals and human populations from health threats in combination with environmental stewardship has not (yet) led to the systematic and sustained allocation of resources for integrated, systems-based health programmes. The session aimed to discuss the development and practical application of OH over time and how its (added) value could be measured. One of the major challenges people are facing when conceptualising the evaluation of OH is the usually complex, interconnected and large scale of the problems that are being tackled by OH programmes or projects. In general, there are few scientific studies published on the evaluation of OH and they are not usually comparable because they are often based on assumptions and expert opinion rather than empirical data making it difficult to explicit these benefits in clear-cut and comparable metrics. This not only constrains decision-making and good resource allocation, but also the innovation of data collection protocols and the development of databases to capture and quantify the value of interdisciplinary approaches. Evaluations should provide information for the contracting institutions on the strengths and weaknesses of their OH initiatives and the impact they achieve, and help OH implementers to assess whether they are making progress towards achievement of the stated goals. Furthermore, there was a call to introduce OH concepts in primary, secondary and tertiary education with the aim to raise awareness and create a natural understanding of systems and their interlinked nature. To enhance the evaluation of OH, it was perceived to be important to create further evaluation capacity by providing training on evaluation of OH/EH and to build stronger links with the community to be able to benefit from community knowledge, approaches and experience.

### Social science, transdisciplinary approach and traditional knowledge systems

This session addressed the role of social science in interdisciplinary and transdisciplinary OH/EH approaches and inclusion of traditional knowledge systems. This is gaining increasing support and attention in different fields of interest, such as health and medicine, ecosystem services, wildlife management, and environmental health. However, in the OH field, integration of social sciences is still to be achieved beyond the restricted role of OH/EH awareness.

The process of how to deal with complexity, also from the scientific perspective, can also be perceived as a social and normative process in itself. Complexity can never be fully grasped and should encourage us to choose what has to be taken into account for understanding and actions. These choices have an important framing effect and are normative in nature, or in the words of Paul Cilliers [15]: *“knowledge is provisional. We cannot make purely objective and final claims about our complex world. We have to make choices and thus we cannot escape the normative or ethical domain.”*

The role of social scientists in the management of OH is also interesting to investigate because their involvement and integration in a disease control team is differently perceived and the risk remains for social scientists to reproduce a rather top-down version of scientific expertise [16]. Public health should also be considered as a social practice because health behaviours are made from a collective decision-making. A clear distinction between disciplinary, multi-, inter- and transdisciplinary research is the level of coordinated collaboration between different forms of knowledge and points of view, and the extent to which such knowledge and viewpoints are integrated [17, 18]. Disciplinary research stays within the limits of one discipline or expert perspective, multidisciplinary research to some extent involves collaboration between different disciplines, be it without major integration of different forms of expertise and knowledge. Interdisciplinary research concerns a coordinated effort of collaboration and integration between disciplines, whereas transdisciplinarity aims at collaboration and integration between academic and non-academic groups, such as on the one hand scientists and on the other hand stakeholder groups, policy experts, the private sector or other practitioners. Often these different forms of collaboration and integration are intermixed which can have a strong impact on how the social reality is going to be described and interventions designed and implemented, often excluding the most vulnerable populations.

### Capacity building and education

The session recognized the initial impetus of OH/EH approaches in the Global South, where they are still crucial given their implications for development, raising diverse challenges from the high diversity of societal and environmental contexts. To prepare the future generation of OH/EH scientists, practitioners and policy makers for the Global South, many education and capacity building initiatives are now developing [19]. While the OH approach often focuses on the link between veterinary and medical sciences, these initiatives, tied to educational or research programs, struggle to take into account ecosystem status and to foster the needed inter- and transdisciplinarity.

The session discussed the challenges posed by OH/EH approaches in settings ranging from small-scale targeted hands-on capacity building in the field, to longer education programs including the context of North-South and South-South cooperation. As the scale of an educational program defines its availability and accessibility, it is hoped that they all adopt a train-the-trainer approach. This session demonstrated a shared concern for building capacities for monitoring, detecting and identifying vectors and pathogens and for educating OH workers on “soft” skills, such as conflict management, leadership, intercultural communication and the abovementioned interdisciplinarity and transdisciplinarity as exemplified by the link between schistosomiasis and dam construction in North-West Senegal [20, 21]. To this end, evidence-based innovative teaching techniques and mixed working groups from different academic backgrounds are recommended. Indeed, too often, field teams from different disciplines work independently, alongside each other on the same study system, and are even forced to rely on different funding sources.

### Feedback on the workshop from European Commission representatives

Delegates from the European Commission attending the workshop insisted that both bottom-up and top-down initiatives are necessary. They underlined the constraint of the vertical, hierarchical structure of administrations at all levels in most countries that has to be overcome to allow a broadly understood OH approach at the policy level. Communication needs to be improved between scientists, policy makers and the public but industry has also to be involved. A clear mission statement is needed and a common language has to be found (e.g. what is meant by prevention?). Also, it was emphasized that social science is too often neglected and that evidence-based values must include social values. This is essential considering how many anthropogenic practices such as land-use changes, food production systems, extraction of natural resources and uncontrolled antimicrobial use can affect zoonotic disease transmission and non-communicable diseases. Many things are done in the EU concerning biodiversity and DG Santé integrates the OH principle. The legal basis is there but the principles are too narrowly understood due to difficulties in communication between actors coming from very different fields. The misunderstanding most probably comes from the fact that the concept is new to most of the population and must be pushed forward by the authorities and the OH community to reach a much broader audience and make it truly transdisciplinary. The officials concluded that we must also use peace moments to prepare for crises, avoid duplication of activities (competition should not dominate collaboration) and collaborate with the non-academic practitioners sector. There is a clear need for prioritization for financial reasons but the importance of context specificity for such prioritization must also be stressed out: depending on a specific period, location, issue, different priorities can be appreciated.

## Conclusions

Participants at the workshop considered of the utmost importance to realize that OH, EH, Planetary Health and related concepts share similar ambitions aimed at the integration of various dimensions and realities that are influencing health. Although these different concepts have different histories within diverse expert contexts, the core message of integration is basically similar. It was preferred to underline commonalities, rather than differences, in order to benefit from complementarity. With regards to OH/EH, the importance of a broader interpretation was emphasised, avoiding a too narrow focus only on links between human and animal health. As further ways forward, stronger and more systematic integration of plant health, food security, agricultural systems and rural development, soil health, well-being, social and cultural drivers and perception of health in the OH approach would also be beneficial. Further, OH/EH may also focus more on benefits of nature to human health. Also OH/EH should take into account more explicitly environmental factors, e.g. climate change effects on the emergence and incidence of infectious diseases and should more prominently take into account various chemical and physical environmental disturbances brought by human activities. It was in any case considered necessary for policy makers to develop clear criteria and indicators for application of OH/EH and related concepts, for the purpose of designing, selecting, financing and evaluating related projects.

An important goal of the OH/EH approach should be to overcome ad hoc reactive actions responding to emerging public health challenges to build pro-active capacity and preparedness, being able to better foresee health risks scenarios thanks to knowledge compiled in integrated databases.

It was also stressed that implementation of OH/EH concepts can benefit from transdisciplinary and iterative processes between policy, science and practice. One should however be careful of creating big OH/EH institutions resulting in building fences rather than creating openness to (new) collaborations. This may be overcome by focusing on open, collaborative networks like Communities of Practice, which are less (institutionally) bound and more flexible, and can be open to newcomers and new ideas and approaches. Such networks should not be limited to scientific experts, but also need to be open to policy experts, local knowledge, practitioners, grass-root organisations and all relevant stakeholders. Inter-and transdisciplinary education at all ages would facilitate this collaborative work and integrative decisions in adult life.

## Abbreviations

ABR: AntiBiotic Resistance
AMR: AntiMicrobial Resistance
CBD: Convention on Biological Diversity
FAO: Food and Agriculture Organisation
NCD: Non Communicable Diseases
NEOH: Network for Evaluation of One Health
OH/EH: One Health / EcoHealth
OIE: Office International des Epizooties
SBSTTA: Subsidiary Body on Scientific, Technical and Technological Advice of the CBD
SWOT: Strengths, Weaknesses, Opportunities and Threats
VBD: Vector Borne Diseases
WHO: World Health Organisation
